# Detection of testosterone based on the interaction between variable regions of antibody heavy chain and light chain

**DOI:** 10.3389/fbioe.2025.1499164

**Published:** 2025-03-20

**Authors:** Guangwei Zhao, Shengshuo Zhang, Yujie Zou, Nan Jia, Liyuan Zheng, Jinhua Dong

**Affiliations:** ^1^ School of Basic Medical Sciences, Shandong University, Ji’nan, China; ^2^ School of Rehabilitation Science and Engineering, University of Health and Rehabilitation Sciences, Qingdao, China; ^3^ School of Life Science and Technology, Shandong Second Medical University, Weifang, China; ^4^ International Research Frontiers Initiative, Tokyo Institute of Technology, Yokohama, Japan

**Keywords:** OS-ELISA, testosterone, phage display, rapid detection, antibody

## Abstract

**Introduction:**

Testosterone is a steroid hormone, which plays a pivotal role in regulating metabolism and protein synthesis in the body. The detection of testosterone is of paramount importance for diagnostic purposes in clinical settings, as well as for monitoring athletes’ physiological parameters and ensuring the integrity of sports competitions.

**Methods:**

Herein, we present a phage display-derived biosensing platform through genetic engineering of the TS77 antibody variable domains. The variable region genes of the heavy and light chains from TS77 antibody were cloned into the pDong1 plasmid and displayed on the phage surfaces through phage display technology. Subsequently, a novel non-competitive open-sandwich ELISA (OS-ELISA) was developed for testosterone detection, leveraging the antigen-induced interaction changes in antibody variable regions.

**Results:**

OS-ELISA based on anti L-chain antibody achieved a limit of detection (LOD) of 2.71 nM and a half-maximal effective concentration (EC50) of 0.22 μM for testosterone detection. Furthermore, the enhanced OS-ELISA platform incorporating purified maltose binding protein fused with V_L_ (MBP-V_L_) and V_H_ phage demonstrated a LOD of 1.07 pM and a wide working range from 1 pM to 10 mM.

**Discussion:**

The OS-ELISA developed in this study exhibits high sensitivity and a broad dynamic range for testosterone quantification, showing significant potential for clinical diagnostics and athlete monitoring applications.

## 1 Introduction

Testosterone (Ide, 2023), a steroidal hormone, plays a critical role in regulating physiological processes and promoting protein synthesis in the body. It is essential for maintaining muscle strength and mass, preserving bone density and strength, improving metabolism, and enhancing overall physical performance. Deviations in testosterone levels have been consistently associated with a range of pathological conditions, such as Polycystic Ovary Syndrome (PCOS) (Hahn et al., 2007), Ovarian Steroid Cell Tumor (OSCT) (Mizoguchi et al., 2014), and Adrenal Cortex Hyperplasia (Ciumas et al., 2009). Furthermore, the detection of testosterone is of vital significance in the physiological assessment of elite athletes. Firstly, testosterone levels serve as a biomarker reflecting an athlete’s athletic capabilities and anabolic metabolism (Wang et al., 2023). Secondly, owing to its potential for performance enhancement, testosterone has been proscribed as a prohibited substance in sports competitions (You et al., 2011). Therefore, the detection of testosterone is of paramount importance for diagnostic purposes in clinical settings, as well as for monitoring athlete performance and ensuring the integrity of sports competitions.

Current methodologies for testosterone detection primarily encompass two categories: those reliant on large-scale equipment and those based on antibody-mediated immunological detection. Within the former, notable examples include the Gas Chromatography-Tandem Mass Spectrometry (GC-MS/MS) approach employed by institutions such as Ghent University in Belgium (Thienpont et al., 1994) and the German Society for Clinical Chemistry and Laboratory Medicine (Siekmann, 1979), as well as the Liquid Chromatography-Tandem Mass Spectrometry (LC-MS/MS) method utilized by organizations like the Health Sciences Authority (Chen et al., 2019), Center for Disease Control and Prevention (Botelho et al., 2013), and National Institute of Standards and Technology (Tai et al., 2007). These methods, while precise, often require skilled operators and complex preprocessing steps.

Alternatively, immunoassay that exploit antigen-antibody interactions, such as the Indirect Competitive Enzyme-Linked Immunosorbent Assay (ic-ELISA) (Jiang et al., 2011) offer simplicity in operation. However, due to the inherent limitations of the competitive format, achieving marked signal changes in the presence of trace analytes necessitates minimizing the amounts of both antibodies and competitive haptens, which leads to the loss in assay sensitivity.

Open Sandwich Enzyme-linked Immunosorbent Assay (OS-ELISA) (Lim et al., 2007) is a novel immunoassay based on the interaction of variable regions of the heavy chain (V_H_) and light chain (V_L_) of the antibody. The principle of OS-ELISA is that the interaction between V_H_ and V_L_ is modulated by the presence of an antigen. V_H_ and V_L_ barely bind to each other in the absence of antigen. When antigen is present, they bind to the antigen to form a trimeric structure, and the signal gradually intensifies as the antigen concentration increases. Based on the principle, a non-competitive immunoassay is established for rapid detection of analytes of interest. OS-ELISA has as already proven successful in the detection of a variety of haptens, including environmental hazards and food additives, such as Microcystin-LR (MCLR) (Chen et al., 2020), Clenbuterol (CLEN) (Cong et al., 2019), Tenuazonic acid (TeA) (Liang et al., 2021) and so on.

In this study, we have developed a novel noncompetitive immunoassay strategy for testosterone. This approach utilizes antigen-driven affinity enhancement, enabling rapid quantification of testosterone. By incorporating the advantages of OS-ELISA, our technique promises to offer a highly sensitive and efficient means for testosterone detection, contributing to both medical diagnostics and athletic performance monitoring.

## 2 Materials and methods

### 2.1 Materials

The TS77 sequence was designed with reference to previously reported sequences (Valjakka et al., 2002). The genes and primers utilized in the experiments were synthesized by Sangon Biotechnology (Shanghai, China). The primers used are summarized in Table 1. *Escherichia coli* DH5α used for gene cloning and plasmid amplification, was purchased from Tsingke Biotech (Beijing, China). *Escherichia coli* TG-1 used for phage display was obtained from GE Healthcare (Tokyo, Japan). *Escherichia coli* BL21-Gold (DE3) pLysS used for expressing the antibody fusion protein, was purchased from Agilent Technologies (La Jolla, California, United States). The restriction endonucleases were sourced from New England Biolabs (Beverly, Massachusetts, United States). Ligation High Ver.2 used for DNA ligation and KOD-Plus-Neo used for PCR, were purchased from TOYOBO (Osaka, Japan). Testosterone was purchased from Aladdin Biochemical Technology (Shanghai, China). Additionally, all other chemicals and reagents were sourced from Vazyme (Nanjing, China) or Sangon Biotech (Shanghai, China).

**TABLE 1:** Primers used in this study.

Primer name	Sequence (5′- 3′)	Length (bp)
M13+	AGGGTTTTCCCAGTCACG	18
M13-	GAG​CGG​ATA​ACA​ATT​TCA​CAC	21
M13Rv	GGA​AAC​AGC​TAT​GAC​CAT​G	19
pHENseq	CTATGCGGCCCCATTCA	17
pDong1CKfor	CGT​ACT​ATG​GTT​GCT​TTG​ACG​TAT	24
pDong1VLback	ATA​TGT​TGC​CAC​CTT​TAT​GTA​T	22
MalE	GGT​CGT​CAG​ACT​GTC​GAT​GAA​GCC	24
M13F	TGTAAAACGACGGCCAGT	18

### 2.2 Construction of pDong1/Fab-TS77

The single chain variable fragment (scFv) gene of TS77 was synthesized and cloned into the pUC57 (Shanghai Sangon Biotech Co., Ltd.). This plasmid was then utilized as a template for the amplification of the scFv gene via polymerase chain reaction (PCR) using KOD Plus Neo and the primers M13+ and M13-. Subsequently, the PCR products were analyzed using agarose gel electrophoresis, and the desired fragments were recovered using a gel extraction kit.

The scFv gene, following its recovery, was subjected to restriction digestion along with the pDong1 (blank) plasmid using *Nco*I and *Xho*I at 37°C for 4 h. The digestion products were analyzed via agarose gel electrophoresis, and the target fragments were purified using a gel extraction kit. Subsequently, the digested plasmid and gene fragment were ligated using Ligation High Vers.2 at 16°C for 2 h. The ligation mixture was then evenly spread onto LBA solid medium (10 g/L tryptone, 5 g/L yeast extract, 5 g/L NaCl, 15 g/L agar, 100 μg/mL ampicillin) for bacterial transformation.

After verifying the clones using PCR with M13Rv and pHENseq primers, the positive clones were chosen, and their plasmids were isolated for sequencing. The positive plasmids containing the scFv gene were further processed with *Sal*I and *Not*I at 37°C for 4 h. The digested products were analyzed, purified, and subsequently ligated again. The ligation mixture was once more spread onto LBA solid medium, and after PCR analysis with primers pDong1VLback and pDong1CKfor, positive clones were picked, their plasmids extracted, and subjected to sequencing to confirm the integrity of the inserted gene. The plasmid with the correct sequence was named pDong1/Fab-TS77.

### 2.3 Phage display of antigen-binding fragment

The plasmid pDong1/Fab-TS77 was transformed into TG-1 competent cells and spread onto 2YTAG solid medium (16 g/L tryptone, 10 g/L yeast extract, 5 g/L NaCl, 15 g/L agar, 100 μg/mL ampicillin and 1% glucose). Single colonies were selected and cultivated in 2YTAG liquid medium (16 g/L tryptone, 10 g/L yeast extract, 5 g/L NaCl, 100 μg/mL ampicillin and 1% glucose) until the OD_600_ reached 0.2. Subsequently, 10^10^ KM13 helper phages (Dong et al., 2009) were added, and the mixture was incubated at 37°C for 1 h. Following incubation, the cells were centrifuged at 5,500 g for 10 min, and the pellet was resuspended in 4 mL of 2YTAGK medium (2YT medium containing 100 μg/mL ampicillin, 50 μg/mL kanamycin, and 0.1% glucose). The culture was then grown at 30°C with shaking at 250 rpm for 20 h.

After cultivation, the cells were centrifuged at 3,300 g for 30 min, and the supernatant was collected. To precipitate the phage, A fifth of the total volume of PEG/NaCl solution (20% polyethylene glycol 6,000, 2.5 M NaCl) was added to the supernatant, followed by incubation on ice for 1 h. Subsequently, the mixture was centrifuged at 5,500 g for 30 min at 4°C. The supernatant was discarded, and the phage pellet was resuspended in 200 μL of phosphate-buffered saline (PBS). To eliminate any remaining cellular debris, the suspension was centrifuged at 11,600 g for 10 min, and the supernatant containing the displaying antigen-binding fragment (Fab) phages was collected.

### 2.4 Detection of testosterone based on OS-ELISA

To eliminate the C_H_1 from the pDong1/Fab-TS77, the plasmid was digested with *Sgr*AI at 37°C for 4 h. Subsequently, the target fragment was recovered using a gel extraction kit and subsequently ligated to itself using Ligation High Ver.2 at 16°C for 2 h. The ligation product was then transformed into TG-1 competent cells, which were evenly spread on 2YTAG solid medium and incubated at 37°C overnight. After confirmation of positive clones through colony PCR with primers M13Rv and pHENseq and sequencing, the positive clone was subjected to expanded culture, and plasmid extraction was performed to obtain pDong1/OS-TS77. The plasmid was transformed into TG-1 competent cells for phage display, with steps similar to those described above. After culture, the supernatant which contained the V_H_ phage and L chains was collected.

For the implementation of OS-ELISA, 100 μL/well of 2 μg/mL rabbit anti-human kappa chain antibody was coated onto microplates and incubated overnight at 4°C. The plates were then blocked with MPBS (PBS containing 2% skim milk) at 25°C for 2 h, followed by 3 washes with PBST (PBS containing 1% Tween 20). Subsequently, a 100 μL mixture of phage solution (containing 10^10^ phages) and antigen dilutions at various concentrations in equal volumes was added and incubated at 25°C for 1 h. After 5 washes with PBST, 0.2 μg/mL of HRP-conjugated anti-M13 antibody was added and incubated at 25°C for 1 h. Following another 5 washes with PBST, the TMBZ solution (100 μg/mL 3, 3′, 5, 5′-tetramethylbenzidine and 0.02 μL/mL H_2_O_2_ in 100 mM NaOAc, pH 6.0) was added for color development, and 50 μL/well of 10% H_2_SO_4_ was used to stopped. After the reaction was terminated, the absorbance values at 450 nm and 630 nm were measured, and the data were analyzed to construct a standard curve.

### 2.5 Preparation of MBP-V_L_ and V_H_ phage

To facilitate the fusion expression of maltose-binding protein (MBP) with the TS77 V_L_ domain, the plasmid pMAL-V_L_ (TS77) was developed based on the pMAL-V_L_ (3A8) plasmid (Chen et al., 2020). The pMAL-V_L_ (3A8) plasmid and the TS77 scFv gene were individually amplified and subsequently digested using *Sal*I and *Not*I restriction enzymes. The digested plasmid and gene fragments were then ligated using Ligation High Ver.2, followed by transformation into DH5α competent cells and even spreading on LBA solid medium. Positive clones were identified through PCR verification using MalE and M13F primers, and selected clones underwent plasmid extraction and sequencing.

The plasmid was then transformed into BL21 (DE3) pLysS competent cells and evenly spread on LBA solid medium. After overnight incubation, a single clone was picked and inoculated into 4 mL LBA liquid medium for cultivation for 12–16 h, which was then transferred to 300 mL LBA liquid medium for expanded growth. When the OD_600_ reached 0.6, isopropyl β-D-1-thiogalactopyranoside (IPTG) with a final concentration of 1 mM was added and then cultured at 25°C with shaking at 250 rpm for 21 h. After a centrifugation at 8,000 g for 20 min at 4°C, the bacterial pellet was resuspended, and the cells were lysed by sonication to obtain a soluble protein solution. This solution was subsequently purified using Ni-NTA Sefinose Resin (Shanghai Sango Biotechnology, Co. Ltd.). To assess the concentration and purity of the purified protein, 10 μL of each sample was analyzed by sodium dodecyl sulfate-polyacrylamide gel electrophoresis (SDS-PAGE) and stained with Coomassie Blue R250.

After phage display of pDong1/OS-TS77, a supernatant containing V_H_ phage and L chain was mixed with a fifth of the total volume of PEG/NaCl and incubated in ice for 1 h, followed by centrifugation at 5,500 g for 1 h at 4°C, and the supernatant was removed. The pellet was resuspended in PBS and centrifuged again, yielding a final supernatant enriched with V_H_ phage.

### 2.6 Detection of testosterone using enhanced OS-ELISA based on MBP-V_L_ and V_H_ phage

For the assay, 100 μL/well of 2 μg/mL MBP-V_L_ was coated onto microplates and incubated overnight at 4°C. 2% MPBS was used to block the wells at 25°C for 2 h, followed by 3 washes with PBST. A mixture of 10^10^ cfu of V_H_ phage solution and a series of concentrations of antigen was added to the wells and incubated at 25°C for 1 h. After washing 5 times with PBST, 0.2 μg/mL of HRP-conjugated anti-M13 antibody was added and incubated at 25°C for 1 h. Following another 5 washes with PBST, the assay was performed as those described above.

### 2.7 Specificity validation of enhanced OS-ELISA

Microplates were coated with 100 μL/well of 2 μg/mL MBP-V_L_ and incubated overnight at 4°C. 2% MPBS was used to block the wells at 25°C for 2 h. After washing 3 times with PBST, 10^10^ cfu of V_H_ phage solution was mixed with different concentrations of testosterone, estradiol, and cortisol. The mixture was added to the wells and incubated at 25°C for 1 h. Then the steps of OS-ELISA was performed as those described above. The data obtained were analyzed to compare the responses generated by testosterone against those of estradiol and cortisol, allowing for the assessment of the specificity of the MBP-V_L_ based enhanced OS-ELISA towards testosterone detection. The results were used to plot standard curves for each hormone, enabling a direct comparison of the sensitivity and specificity of the assay towards testosterone versus potential cross-reactive hormones such as estradiol and cortisol.

### 2.8 Molecular docking and prediction of amino acid interaction sites

To evaluate the binding affinity and interaction patterns between TS77 and testosterone, we used Autodock Vina 1.2.2 (Zuo et al., 2005), a computer-aided protein-ligand docking software. The molecular structure of testosterone was retrieved from the PubChem compound database (https://pubchem.ncbi.nlm.nih.gov/). The variable region sequence of the TS77 antibody was predicted using Swiss-Model (http://swissmodel.expasy.org/). Initially, the antibody and small molecule files were prepared by converting them to the PDBQT format, excluding all water molecules, and appending polar hydrogen atoms. The grid box was centered to encompass the domain of each protein structure while accommodating the movement of the free molecule. The docking pocket was configured as a 30 Å × 30 Å×30 Å cubic box with a grid spacing of 0.05 nm. The molecular docking was performed using Autodock Vina 1.2.2 (http://autodock.scripps.edu/), and the results were utilized for model visualization.

### 2.9 Data analysis

The data obtained from OS-ELISA were analyzed using Prism 9 (GraphPad Software, San Diego, CA, United States). The dose–response curves were fitted with a four-parameter equation as follows:

$$y=a+\frac{b-a}{{\left(1+\frac{x}{c}\right)}^{d}}$$

where:

*y* is the response at concentration *x*,
*a* is the maximum response (in arbitrary units, a.u.),
*b* is the slope factor (dimensionless),
*c* is the half-maximal effective concentration (in picoMolar, pM),
*d* is the minimum response (in arbitrary units, a.u.).

The EC_50_ was obtained from Nonlin fit results of dose–response curves. The LOD for each assay was obtained by adding the estimated antigen concentration that showed the mean blank value to 3 times the standard deviation for the OS-ELISA.

## 3 Results

### 3.1 Phage display of TS77 Fab

**FIGURE 1:**
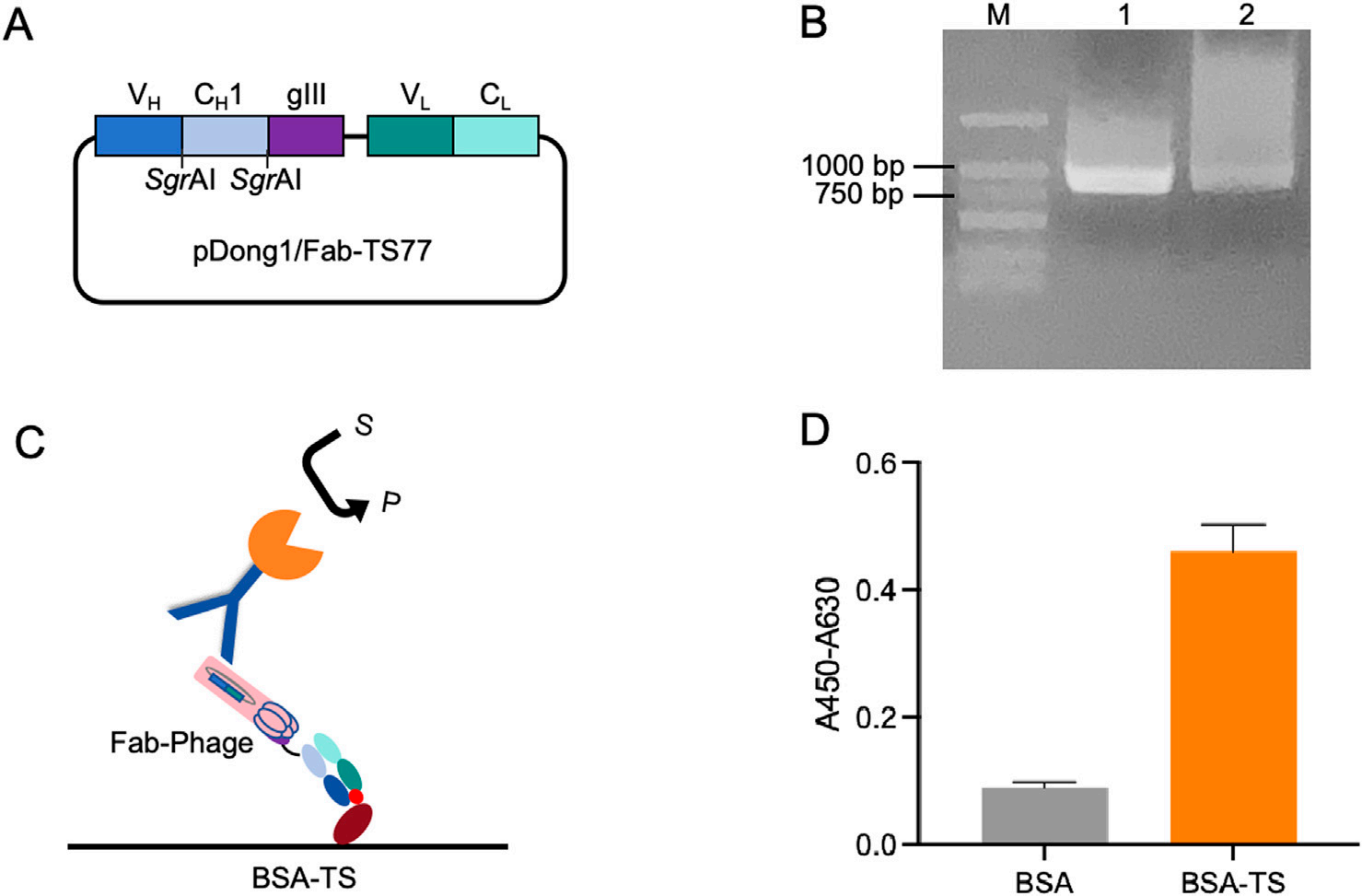
Phage display of TS77 antigen-binding fragment. **(A)** Schematic of the pDong1/Fab-TS77. **(B)** Agarose gel electrophoresis analysis of TS77 scFv gene. M: DNA marker. **(C)** Schematic of the ELISA. **(D)** ELISA analysis of the antigen binding activity of phage-displayed Fab. Error bars represent standard deviation (SD) of triplicate samples.

To validate the antigen-binding activity of the TS77 Fab expressed via phage display, we constructed the pDong1/Fab-TS77 Figure 1A. The Fd was fused with the phage pIII protein for expression, and the *Sgr*AI site was added to both ends of the C_H_1 domain, while the L chain was expressed independently. Through PCR amplification of the TS77 scFv, distinct bands were observed between 750 and 1,000 bp after agarose gel electrophoresis, indicating successful amplification of the TS77 scFv Figure 1B. Following digestion with restriction endonucleases, the V_H_ and V_L_ genes were obtained. These were subsequently cloned into the pDong1-blank vector, resulting in the acquisition of pDong1/Fab-TS77.

After phage display, the TS77 Fab phage was obtained with a titer of 10^13^ cfu/mL. To verify the activity of the phage-displayed TS77 Fab, we coated microplates with BSA and BSA-TS separately and identified its antigen-binding activity using ELISA Figure 1C. As shown in Figure 1D, the absorbance of the BSA-TS was significantly higher than that of the negative control BSA, indicating that the phage-displayed TS77 Fab exhibited good binding activity towards testosterone.

### 3.2 Detection of testosterone by OS-ELISA

**FIGURE 2:**
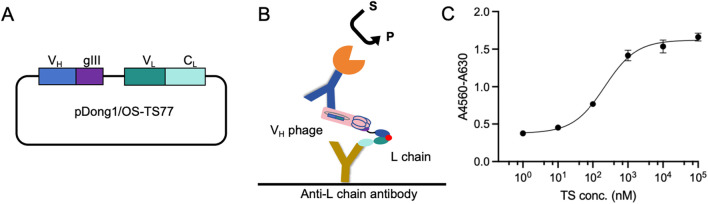
Open sandwich phage ELISA. **(A)** Schematic of the pDong1/OS-TS77. **(B)** Schematic of the OS-ELISA. **(C)** Dose-response curve for the detection of testosterone concentration. Error bars represent standard deviation (SD) of triplicate samples.

Subsequently, we removed the *Sgr*AI restriction sites from both ends of C_H_1 to obtain the pDong1/OS-TS77 plasmid Figure 2A. Following phage display, the V_H_ was presented on the phage surface, while the V_L_ remained free in the culture medium due to the loss of the C_H_1-C_L_ dimerization. Utilizing an Anti-L chain antibody, we directly employed the culture medium containing both the L chain and V_H_ phage for testosterone detection. The schematic of the OS-ELISA is depicted in Figure 2B. The OS-ELISA results showed an increase in absorbance with rising testosterone concentrations, indicating a dose-dependent relationship Figure 2C. The limit of detection (LOD) was calculated to be 2.71 nM, and the half-maximal effective concentration (EC_50_) was 0.22 μM. These results demonstrate that the OS-ELISA method is capable of detecting testosterone effectively.

### 3.3 Enhanced OS-ELISA utilizing MBP-V_L_ and V_H_ phage

**FIGURE 3:**
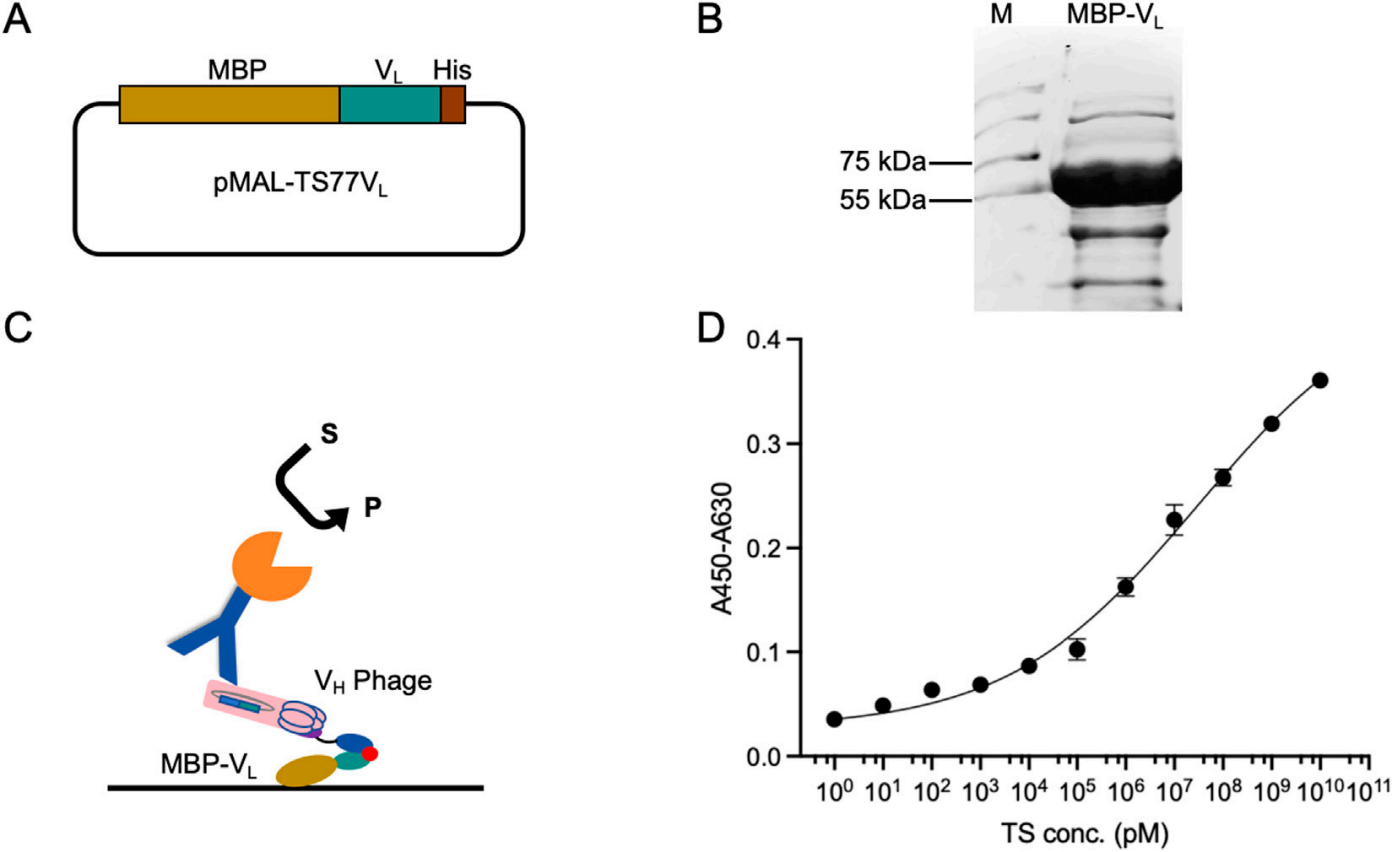
Enhanced OS-ELISA using purified phage displayed V_H_ and MBP-V_L_ fusion protein. **(A)** Schematic of the pMAL-TS77V_L_. **(B)** Schematic of the enhanced OS-ELISA. **(C)** SDS-PAGE analysis of MBP-V_L_ with Coomassie Blue staining. **(D)** Dose-response curve for the detection of testosterone concentration. Error bars represent standard deviation (SD) of triplicate samples.

To further enhance the detection sensitivity and range, we fused the V_L_ with MBP to obtain MBP-V_L_. Fusion with MBP enhances the stability, expression level, and coating efficiency of V_L_, while mitigating signal loss in multi-step binding processes during OS-ELISA. The TS77 V_L_ was cloned into the pMAL-blank vector to generate pMAL-TS77V_L_ Figure 3A. The plasmid was expressed in BL21 (DE3) pLysS. The purity of the expressed MBP-V_L_ was confirmed by SDS-PAGE, which revealed a distinct protein band near 55–75 kDa Figure 3B, consistent with the predicted size of 60.7 kDa for MBP-V_L_, indicating successful expression.

OS-ELISA for testosterone detection was performed using MBP-V_L_ and V_H_ phage Figure 3C. As shown in Figure 3D, a dose-dependent relationship was observed between the absorbance and testosterone concentration, with significant improvements in sensitivity and detection range. And the wide detection range is from 1 pM to 10 mM. Besides, the LOD was lowered to 1.07 pM, and the EC_50_ was 28.7 μM. This enhanced OS-ELISA exhibited a 2000-fold decrease in LOD, resulting in a substantial improvement in sensitivity. In comparison, commercial testosterone ELISA kits typically have LOD ranging from 100 to 200 pM, suggesting that the enhanced OS-ELISA based on MBP-V_L_ and V_H_ phage possesses ultra-high sensitivity.

**FIGURE 4:**
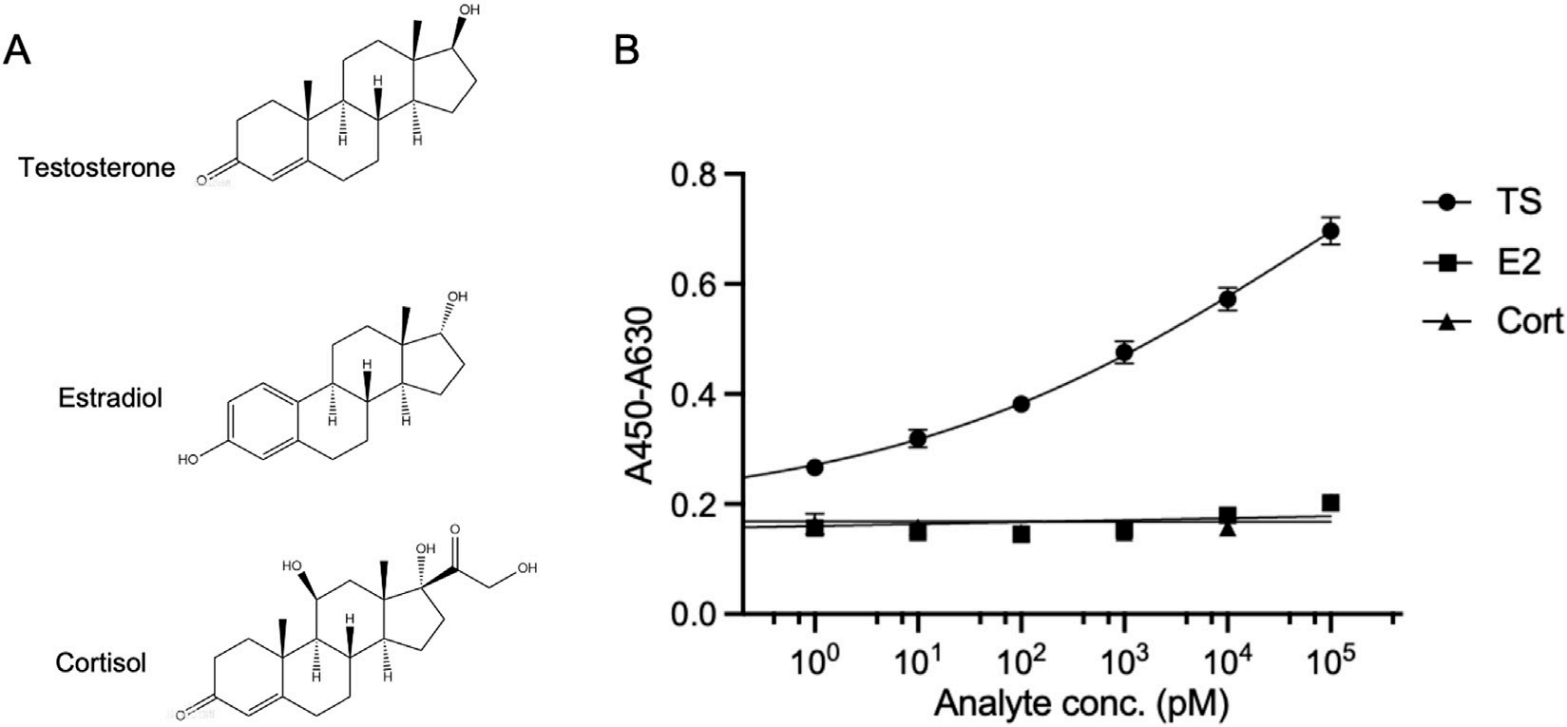
Cross-reactivity of OS-ELISA with structural analogues. **(A)** Structures of testosterone, estradiol and cortisol. **(B)** Dose-response curve of the analyte detection. Error bars represent standard deviation (SD) of triplicate samples.

To further validate the specificity of the OS-ELISA for testosterone detection, we performed simultaneous detection of estradiol (Waifalkar et al., 2022) and cortisol (Hameed et al., 2013), which has a similar structure to testosterone Figure 4A, using the enhanced OS-ELISA. The absorbance of the reaction system was measured in the presence of a series of concentrations of these analytes. As shown in Figure 4B, the OS-ELISA exhibited distinct differences in response to the different analytes. With increasing concentrations of testosterone, the absorbance displayed a dose-dependent increase. In contrast, no significant increase in absorbance was observed with increasing concentrations of estradiol or cortisol. These results suggest that the OS-ELISA detection method exhibits strong specificity towards testosterone.

### 3.4 Molecular docking and binding site analysis

**FIGURE 5:**
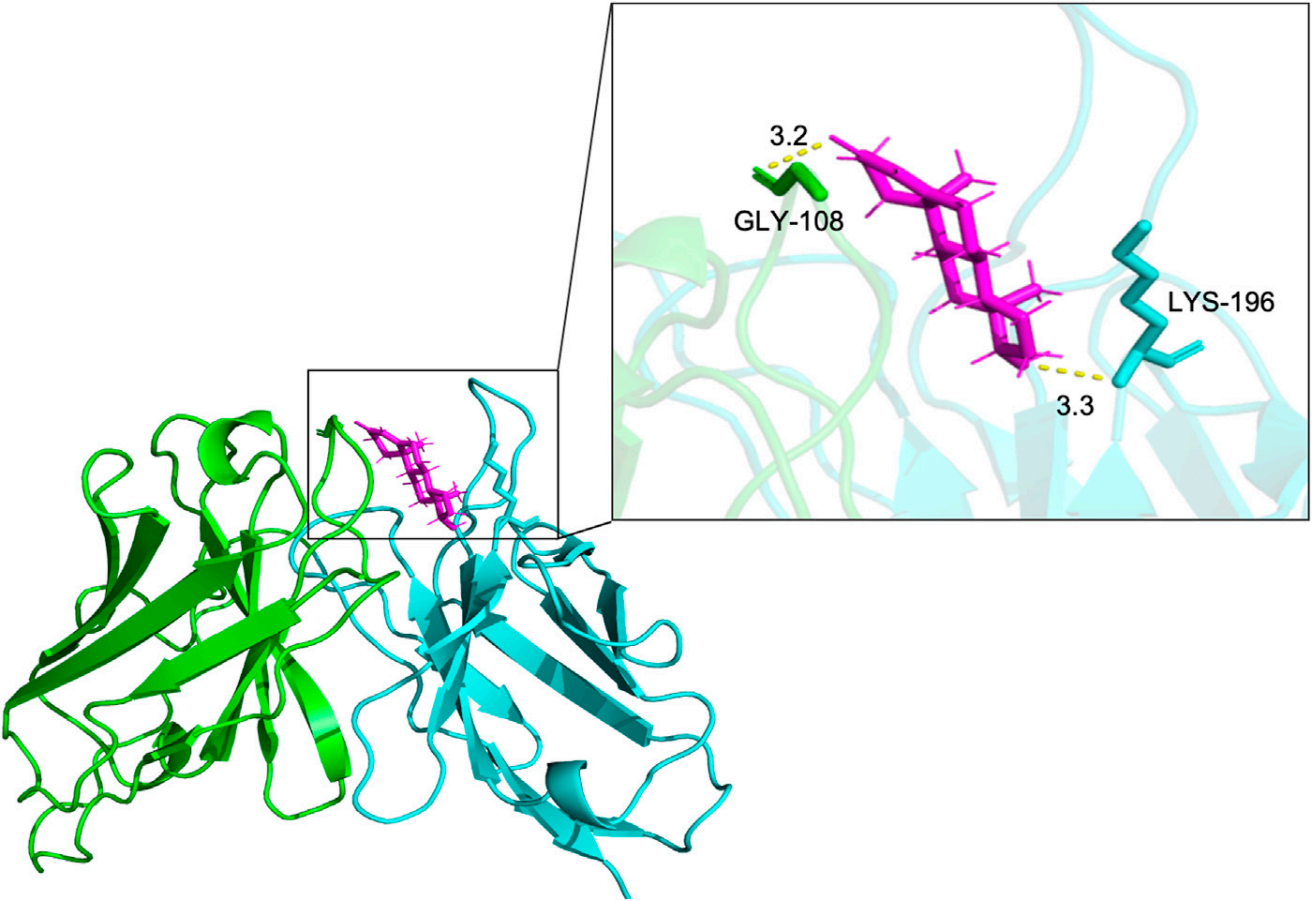
The docking results of antibody V region and testosterone. Key amino acids in V_H_ and V_L_ were presented by different colors of sticks. The results were presented in form of PyMOL. Hydrogen bonds are shown as yellow dotted lines.

To analyze the binding site of TS77 with testosterone, molecular docking analysis was performed. The binding position and interactions between the TS77 scFv and testosterone were obtained using AutoDock Vina v.1.2.2. The binding free energy was calculated to be −8.275, indicating a highly stable binding. Notably, the GLY-108 of V_H_ and the LYS-196 of V_L_ were identified as key residues forming visible hydrogen bonds with testosterone Figure 5. This suggests that the presence of testosterone facilitates the formation of a more stable trimer consisting of these three components, which is crucial for the successful establishment of the OS-ELISA.

## 4 Discussion

Testosterone, as a steroid hormone, plays a pivotal role in physiological regulation and protein synthesis, making it a key reference in disease diagnosis and functional assessment of athletes (Mulhall et al., 2018). Conventional instrumental methods for testosterone detection primarily include GC-MS/MS, LC-MS/MS, among others. These methodologies exhibit high sensitivity, along with a broad linear range. However, they necessitate specialized technical expertise from operators and impose stringent requirements on experimental instruments and conditions.

In this study, we have developed a non-competitive immunoassay method specifically for testosterone, which differentiates itself from traditional immunoassays that employ full-length antibody. The OS-ELISA is a detection method utilizing recombinant antibody fragments, V_H_ and V_L_ domains (Ueda et al., 1996). This approach circumvents the limitation of epitope quantity and is capable of detecting haptens with molecular weights less than 1,000 Da (Suzuki et al., 2000). The efficacy of ic-ELISA is often dictated by the affinity of the antibodies used, thereby restricting its sensitivity and detection range. Conversely, OS-ELISA offers non-competitive detection of small molecules, with enhanced sensitivity and an expanded detection range (Gonzalez-Techera et al., 2007).

The success of this detection method hinges critically on the enhancement of the affinity between V_H_ and V_L_ in the presence of the antigen, thereby necessitating a low inherent affinity between V_H_ and V_L_ (Islam et al., 2010). The pDong1 phage display system (Dong et al., 2009) employed in this study not only display antibody fragments on the phage surface but also facilitates the assessment of V_H_-V_L_ interactions. After validating the antigen-binding activity of the Fab fragment through phage display, we utilized the *Sgr*AI sites flanking the C_H_1 domain in pDong1/Fab-TS77 to excise C_H_1. Since Fd is fused to the phage pIII protein, while the L chain exists as a separate open reading frame, excision of C_H_1 disrupts their dimerization, allowing the light chain to exist independently in solution. Subsequently, the results of OS-ELISA can be used to evaluate the interaction between V_H_ and V_L_. It also provides reference for the development of another immunoassay, Quenchbody (Q-body) (Dong and Ueda, 2021).

MBP exhibits excellent solubilizing properties and is frequently incorporated into prokaryotic expression systems as a solubilizing tag. In this study, the fusion of V_L_ with MBP enhanced the sensitivity of OS-ELISA. Firstly, in the light chain antibody detection system, the ratio of V_H_ to V_L_ is fixed and cannot be altered. By preparing MBP-V_L_ separately, the sensitivity can be improved through optimized adjustment of the V_H_-to-V_L_ ratio. Secondly, the fusion with MBP increased the expression level of V_L_, facilitating subsequent modifications and transformations. The use of MBP-V_L_ not only augmented the quantity of V_L_ but also, due to its higher molecular weight, exposed more V_L_-binding sites during the coating process, thereby enhancing the sensitivity of the assay.

Molecular docking was performed to simulate the binding position and interaction sites of the TS77 variable region with testosterone. As expected, testosterone forms hydrogen bonds with amino acids on both V_H_ and V_L_. When V_H_ and V_L_ bind to testosterone, a stable trimer is formed among the three components, reinforcing the potential of this approach for highly sensitive and specific detection.

A comparison of OS-ELISA with previously published systems for testosterone detection is shown in Table 2. Compared to the traditional ic-ELISA method, OS-ELISA exhibits a broader detection range and higher sensitivity. It was reported that the reference range of serum total testosterone in male adults showed to be 2.01–7.50 ng/mL (6.97–26.00 nM) (Iwamoto et al., 2004). In this study, OS-ELISA based on anti L chain antibody achieved a LOD of 2.71 nM and an EC_50_ of 0.22 μM. Furthermore, the enhanced ELISA with a detection range from 1 pM to 10 mM utilizing MBP-V_L_ achieved an even lower LOD of 1.07 pM, and an EC_50_ of 28.7 μM, significantly outperforming commercial ic-ELISA kits in terms of sensitivity. Additionally, OS-ELISA demonstrated high specificity when compared against two structurally similar hormones. This methodology has the potential to offer a novel approach for the prevention, rapid diagnosis, prognosis of testosterone-related diseases, as well as the monitoring of physical condition and doping detection in elite athletes.

**TABLE 2:** Different methods for measurement of testosterone.

Method	Detection range	LOD	Ref.
OS-ELISA	1 pM-10 mM	1.07 pM	This work
Amperometric	10–500 ng/mL	16.72 pg/mL	Bulut et al. (2020)
Electrochemical impedance spectroscopy	0.045 ng/mL	0.05–5 ng/mL	Li et al. (2016)
Indirect competitive enzyme IA	0.03–30 ng/mL	20 pg/mL	Wang et al. (2016)
Chemiluminescent IA	0–25 ng/mL	0.45 ng/mL	Hu et al. (2017)
Proof-of-concept assay	0.39–1.56 μM	0.29 μM	Cánovas et al. (2022)
Liquid Chromatography-Tandem Mass Spectrometry	7.3 pM–45.1 nM	9.71 pM	Wang et al. (2014)

## 5 Conclusion

In this study, we present the development of a novel non-competitive OS-ELISA for the sensitive and specific detection of testosterone. This innovative approach exploits the enhanced interaction between antigen-driven antibody V_H_ and V_L_, offering a significant advancement in immunoassay technology. Initially, a foundational OS-ELISA platform was established using an expression mixture of V_H_ and dissociated V_L_ derived from phage display. Building on this, we further optimized the assay by incorporating V_H_ and MBP-V_L_, which markedly improved both sensitivity and specificity. The resulting OS-ELISA demonstrates exceptional performance, positioning it as a highly reliable and practical analytical tool for testosterone detection. With its robust design and superior performance, this method holds great promise for applications in clinical monitoring and *in vitro* diagnostics, offering an alternative to conventional immunoassays.

## Data Availability

The original contributions presented in the study are included in the article/supplementary material, further inquiries can be directed to the corresponding author.
